# Advances in Collaborative Practice for Patients With Head and Neck Cancers

**Published:** 2017-04-01

**Authors:** Robert Haddad, Jason Glass

**Affiliations:** Dana-Farber Cancer Institute, Boston, Massachusetts

## Abstract

Head and neck cancer care is complex, and there are limited treatment options; over the past decade only one new therapy received FDA approval. However, as in other tumor types, the landscape is changing with the recent emergence of newer targeted therapies and immunotherapies.

"By the year 2020, there will be more head and neck cancers linked to human papillomavirus [HPV] in the United States than cervical cancer," according to Robert Haddad, MD, a medical oncologist at the Dana-Farber Cancer Institute, Boston. "We are seeing a change in the epidemiology of this disease in the United States and Western Europe," he continued, with "HPV infection now being the number one cause of head and neck cancer in the oropharynx in the United States."

At JADPRO Live at APSHO 2016, Dr. Haddad was joined by Jason Glass, ACNP-BC, also of Dana-Farber Cancer Institute, to discuss the risk factors for head and neck cancer, focusing on HPV infection; the treatment approaches ranging from surgery to chemoradiotherapy; and the emerging role of immunotherapeutic agents for advanced head and neck tumors as well as practical strategies for managing their unique side effects.

## RISK FACTORS

Traditionally, head and neck cancer has been strongly associated with tobacco use and alcohol consumption. "Fifteen years ago, almost every patient with head and neck cancer used to be a heavy drinker or smoker," Dr. Haddad stated. That is no longer the case. Smoking-related cancers appear to be declining, whereas HPV-related oropharyngeal cancers are increasing. Many patients now are nonsmokers with a low level of alcohol consumption.

HPV infection is not only the main cause of cervical cancer in women and anal cancer in both men and women; it is now the number one cause of oropharyngeal cancer in both men and women, although more often in men. "Today, the typical patient is a 45- or 50-year-old man, nonsmoker, nondrinker, with a neck node (oropharyngeal primary), and HPV infection," Dr. Haddad indicated.

HPV 16 is the viral subtype in the vast majority of patients with HPV-positive head and neck cancer. It is increasing in incidence and appears to be associated with multiple sexual partners and high-risk sexual practices (Chaturvedi Engels, Anderson, & Gillison, 2008; Fakhry & Gillison, 2006).

Dr. Haddad encouraged clinicians to determine the HPV status of patients with head and neck cancer. "At Dana-Farber Cancer Institute, checking for HPV status is standard of care for all oropharyngeal cancers, and that is how it should be everywhere," he suggested.

This is important because HPV-positive cancer seems to convey a much more favorable prognosis, with cure rates of 80% to 90%, vs. 50% to 60% with non-HPV cancer (Ang et al., 2010). "If you have to get this cancer, it is better to get it from the HPV virus than from smoking," Dr. Haddad commented.

In the phase III RTOG 0129 trial, Ang and colleagues (2010) compared survival outcomes by HPV status in patients with advanced squamous cell carcinoma of the head and neck treated with concomitant chemoradiotherapy or accelerated-fractionation radiotherapy. They found that 3-year overall survival was better for those with HPV-positive tumors than for those with HPV-negative tumors (82.4% vs. 57.1%; p < .001). "Outcome for these [HPV-positive] patients is excellent. They do very well with appropriate therapy," he said.

Consequently, de-intensification of therapy for patients with HPV-positive tumors has emerged as an important topic. "Because these patients do so well, an area of research currently in our field is can we de-intensify therapy and maintain this high cure rate?" he asked.

## TREATMENT APPROACHES

Initial treatment of head and neck cancer depends on the primary site. For anterior-location tumors (e.g., of the oral cavity, floor of the mouth, cheeks, or lips), surgery is the first approach. For tumors with a posterior location (e.g., oral pharynx, tonsils, tongue, hypopharynx), the initial treatment is chemoradiotherapy to preserve organs, Dr. Haddad said.

Treatment is then determined by the extent of disease (Table 1). For stage 1 or 2 tumors, the unimodality approach consists of surgery or radiotherapy. Oncologically adequate resection with clear surgical margins is the general principle of surgery. Since this surgery is complex, often requiring reconstruction, head and neck cancers should be treated by an experienced head and neck surgeon in an academic center, he maintained. "Achieving a negative margin has a big effect on survival," he added.

**Table 1:**
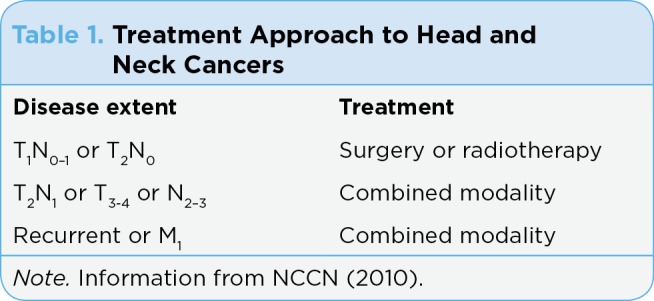
Treatment Approach to Head and Neck Cancers

Radiation therapy is the mainstay of treatment for head and neck cancer, and will be required for almost all patients with stage 3 or 4 disease. Combined-modality therapy (surgery and/or radiation therapy or chemoradiotherapy) is indicated for recurrent or metastatic tumors. According to Dr. Haddad, concurrent chemoradiotherapy is the standard approach to head and neck cancer.

Chemoradiotherapy can be given concurrently or sequentially, and the debate over the therapeutic sequence continues (Pignon, Bourhis, Domenge, & Designe, 2000; Monnerat, Faivre, Temam, Bourhis, & Raymond, 2002). Meta-analyses have shown that outcomes in patients with squamous cell carcinoma of the head and neck were improved with the addition of radiation to chemotherapy (Pignon et al., 2000).

According to Dr. Haddad, the current standard of care in head and neck cancer is cisplatin and radiation therapy. "It is important to use cisplatin in head and neck cancer and not other agents," Dr. Haddad emphasized. For patients who are not candidates for cisplatin, alternatives include carboplatin, paclitaxel, or cetuximab (Erbitux). Potential approaches to improve on chemoradiotherapy include the addition of induction chemotherapy and accelerated-fractionation of radiotherapy.

The use of sequential chemoradiotherapy (giving induction chemotherapy before radiotherapy) was evaluated in the TAX 324 trial (Posner et al., 2007). In this phase III trial of more than 500 patients with mostly locally advanced, nonmetastatic tumors, the triplet of docetaxel, cisplatin, and fluorouracil (5-FU) significantly improved overall and progression-free survival compared with cisplatin and 5-FU. Based on these trial results, this triplet has become the standard induction regimen. Based on the occurrence of more neutropenia and febrile neutropenia with the three drugs, growth factors could be considered.

Another option for recurrent disease without surgery or radiation therapy is the use of platinum-based chemotherapy plus cetuximab (Vermorken et al., 2008). The triplet of cisplatin or carboplatin, 5-FU, and cetuximab improved survival by almost 3 months compared with the same treatment without cetuximab in patients with recurrent or metastatic squamous cell carcinoma (10.1 months vs. 7.4 months; *p* = .04).

## ROLE OF IMMUNOTHERAPY

Immune checkpoint inhibitors (i.e., inhibitors of programmed cell death protein 1 [PD-1]) have had a major impact on the treatment of many solid tumors and now "have found their way into head and neck cancer," noted Dr. Haddad. Pembrolizumab (Keytruda) received US Food and Drug Administration (FDA) approval in August 2016 for treatment of platinum-refractory recurrent or metastatic squamous cell head and neck cancer that progressed after chemotherapy, and the FDA is currently considering nivolumab (Opdivo) in this population. Ongoing studies are evaluating these agents in the platinum-sensitive or first-line settings for this malignancy.

The study on which approval of pembrolizumab was based is the KEYNOTE-012 trial (Mehra et al., 2016). In this heavily pretreated population of patients with squamous cell head and neck tumors, the response rate to pembrolizumab was 18% (slightly higher in HPV-positive patients). The overall survival rate was 58% at 6 months and 38% at 12 months.

Nivolumab is under evaluation in the CheckMate 141 study in the same recurrent or metastatic squamous cell head and neck cancer population (Ferris et al., 2016). Compared with the investigator’s choice of treatment, nivolumab yielded better overall survival (7.5 vs. 5.1 months). "This is the first phase III study to show an improvement in survival with nivolumab compared with chemotherapy," stated Dr. Haddad. He added that patient-reported outcomes are also favorable, with appetite loss, fatigue, and dyspnea remaining stable with nivolumab but deteriorating with chemotherapy (Harrington, 2016).

## MANAGING SIDE EFFECTS OF IMMUNOTHERAPY

The PD-1 inhibitors represent a new standard-of-care option for patients with recurrent or metastatic squamous cell head and neck cancer after platinum-based therapy. However, to benefit from these important new agents, patients must be able to cope with their unique side effects and remain on therapy, according to Mr. Glass, who shared the collaborative practice approach used at Dana-Farber Cancer Institute.

The head and neck oncology team includes the medical oncologist, radiation oncologist, surgeon, nurse practitioner or physician assistant, and program nurse. The treatment support team consists of a host of specialists, such as the primary nurse, nutritionist, speech and language pathologist, and social worker. Specialists in pain and palliative care, dermatology, oral medicine, and psychiatry round out the collaborative care team.

"It sounds like a lot of cooks in the kitchen, and sometimes it can be overwhelming," Mr. Glass acknowledged, "but it works seamlessly, and people can be pulled in at a moment’s notice." The goal is to improve outcomes for patients by preventing treatment delays and keeping patients out of the emergency room.

Before starting patients on immunotherapy, advanced practitioners should repeat the medical history screening for autoimmune disorders or conditions requiring immunosuppression; perform laboratory tests (baseline thyroid, liver, creatinine) and repeat them periodically throughout and after therapy; and discuss reproductive concerns, as immunotherapy can cause fetal harm.

Common side effects of immunotherapy include fatigue, pruritus, diarrhea, rash, dyspnea, decreased appetite, and constipation; rarer adverse events consist of infusion-related reactions, neurologic effects, and immune-mediated responses (e.g., pneumonitis, dermatitis, colitis, and hepatitis; Table 2).

**Table 2:**
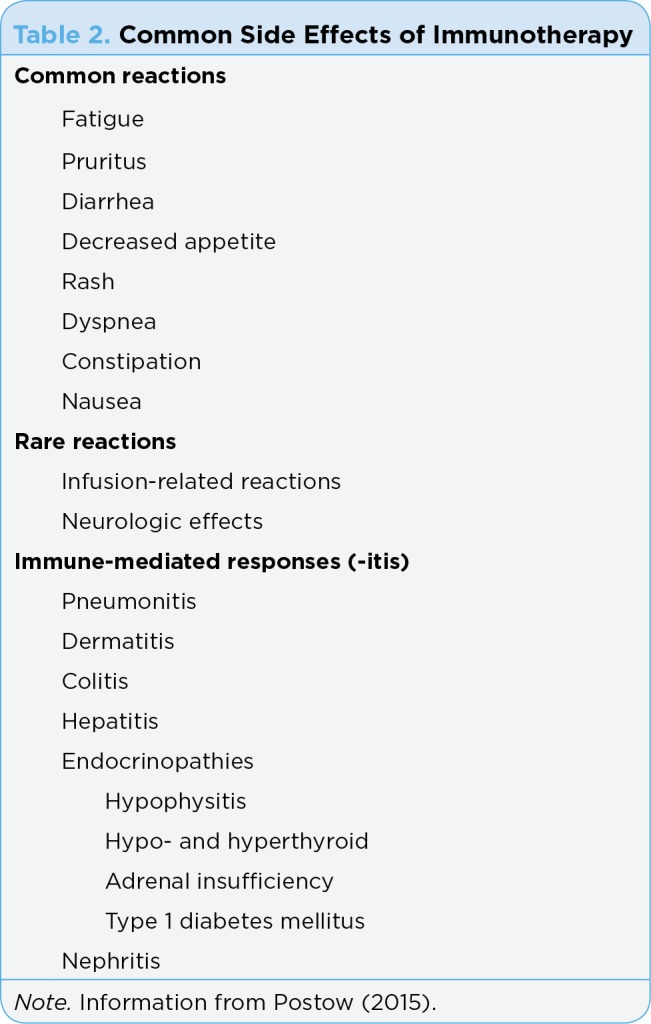
Common Side Effects of Immunotherapy

Mr. Glass emphasized the importance of discussing potential side effects with patients before treatment. "This allows them to be part of the conversation," he said. Generally, patients tolerate these agents well and have a fairly low risk of serious side effects. "Still, they do need to be monitored very closely," Mr. Glass added.

## RARER ADVERSE EVENTS

Mr. Glass described those adverse events that are rare but can be problematic as: "the ones that get in the way of treatment, delaying it or permanently stopping it." Pneumonitis is the most complicated to deal with. Symptoms such as new or worsening cough, shortness of breath, chest tightness, or pain can signal many possible clinical scenarios. In addition, "patients are at high risk of aspiration, so it could be an infection," Mr. Glass said. The differential diagnosis also includes pneumonia and pulmonary embolus.

The workup includes computed tomography of the chest; clinicians should consider an infectious disease consultation. For grade 1 pneumonitis, advanced practitioners should consider holding treatment; for grade 2 pneumonitis, treatment can be held and corticosteroids started; for grade 3/4 pneumonitis, treatment should be permanently discontinued, steroids started, and nonsteroidal immunosuppression considered (Merck, 2014).

Dermatitis occurs in about 30% of patients, although Mr. Glass indicated the frequency seems greater than this in his own practice. "Typically, some patients may still have some radiation effects, and they may already be coming in with impaired skin," he cautioned.

Key management steps are to minimize the risk by starting early with gentle moisturizers, minimizing sun exposure, and avoiding tight-fitting clothing (Dadu, Zobniw, & Diab, 2016). For grade 1/2 reactions, treatment can be continued and antihistamines and topical steroids started; for grade 3 reactions, treatment should be held, the patient referred to a dermatologist, and oral steroids started; for grade 3/4 reactions, treatment should be stopped, the patient referred to a dermatologist, and oral steroids initiated (Merck, 2014). Higher-grade effects are uncommon.

Colitis is another rare side effect of immunotherapy. The key to managing inflammation of the colon is early treatment of gastrointestinal symptoms, which "leads to a more rapid response," Mr. Glass said. It is also important to rule out bacterial, viral, or parasitic infections (Pernot, Ramtohul, & Taieb, 2016).

For a grade 1 reaction, treatment should be continued and symptoms managed; for a grade 2 reaction, treatment should be held, symptoms treated, and corticosteroids started (if symptoms last more than 5 days); grade 3 colitis is treated the same as grade 2 colitis, but endoscopy should be considered; for grade 4 colitis, therapy should be permanently discontinued, high-dose steroids started, and endoscopy considered (Merck, 2014).

Finally, evidence of hepatitis (inflammation of the liver and elevation of liver function tests [LFT]) requires attention during immunotherapy. In addition, an obstruction, alcohol abuse, and use of statins or acetaminophen should be considered as causes.

For grade 1 reactions, treatment should be continued and LFTs monitored; for grade 2 reactions, treatment should be held, LFTs monitored every 3 days, and corticosteroids started; for grade 3/4 reactions, therapy should be permanently discontinued, high-dose steroids started, and the patient referred to a gastroenterologist.

It appears that anti–PD-1 antibodies can affect any organ system, and thus all symptoms should be considered as potentially associated to anti–PD-1 therapy, according to Hofmann and colleagues (2016). In addition to the skin, gastrointestinal tract, liver, and endocrine system, the musculoskeleton, heart, and eyes can also be affected by immunotherapy.

Generally, immunotherapy is well tolerated, but some of its unique toxicities can be life-threatening. Thus, early identification and close monitoring are key to managing these adverse events. Steroids, with a long and slow taper, represent a mainstay in managing immune-related side effects, and multidisciplinary team care is essential. Mr. Glass concluded: "The treatment of head and neck patients is very challenging, and putting them on immunotherapy does not make that any easier."
